# Sexual selection, genomic evolution and population fitness in *Drosophila pseudoobscura*

**DOI:** 10.1098/rspb.2024.2744

**Published:** 2025-04-02

**Authors:** Stewart Leigh, Peter Thorpe, Rhonda R. Snook, Michael G. Ritchie

**Affiliations:** ^1^ Centre for Biological Diversity, School of Biology, University of St Andrews, St Andrews, Fife, UK; ^2^ The Data Analysis Group, School of Life Sciences, University of Dundee, Dundee, UK; ^3^ Department of Zoology, Stockholms Universitet, Stockholm, Sweden

**Keywords:** sexual selection, sexual conflict, mutational load, distribution of fitness effects

## Abstract

Sexual selection shapes the genome in unique ways. It is also likely to have significant fitness consequences, such as purging deleterious mutations from the genome or conversely maintaining genetic load in a population via sexual conflict. Here, we examined what the influence of sexual selection has on genomic variation potentially underlying population fitness using experimentally evolved *Drosophila pseudoobscura* populations. Sexual selection was manipulated by keeping replicate lines in elevated polyandry or strict monogamy for approximately 200 generations followed by individual-based sequencing. Using pi (*π*), fixation index (*F*_st_)and recombination rate measures, we confirmed signatures of selection were not dispersed but mainly localized to the third and X chromosome. Overall mutational load was similar between lines but our analysis of the distribution of fitness effects revealed considerable variation between lines and chromosomes. Furthermore, we found that the distribution of transposable elements differs between the lines, with a higher load in monogamous lines. Our results suggest that complex interactions between purifying selection and sexual conflict are shaping the genome, particularly on chromosome 3 and the sex chromosome; sexual selection influences divergence across chromosomes but in a more complex way than proposed by simple ‘purging’ of deleterious loci.

## 1. Introduction

Sexual selection is responsible for shaping and maintaining many aspects of a species' phenotype. Previous work has identified how sexual selection can influence specific traits that have a role in mating and fertilization success, such as the production and composition of seminal fluid proteins [1], courtship behaviours [2] and secondary sexual characteristics [3,4]. Yet the evolutionary consequences of sexual selection for genomic structure and variation remain understudied [5]. This can be partly ascribed to difficulty in untangling sexual selection from other evolutionary drivers such as natural selection and drift. One recent way of clarifying differences due to sexual selection is via experimental evolution studies manipulating the intensity of sexual selection [6].

The effect of sexual selection on genomic variation could influence a population’s fitness. Mutations are introduced into a population each generation, most of which are predicted to be neutral or deleterious [7]. Purifying selection is the process whereby deleterious mutations are removed, preventing them from accumulating and causing a decrease in population fitness and increase in extinction risk [8]. Sexual selection has been proposed as one mechanism that can contribute to purifying selection, as female choice or male–male competition can select against males with a higher genetic load, helping to purge the population of deleterious alleles [9,10]. This may be particularly effective if secondary sexual characteristics are condition-dependent indicators of ‘good genes’ as proposed by the genic capture hypothesis [11] or at least a lack of ‘bad genes’ [12,13]. However, we do not yet know the extent of the contribution of sexual selection to purifying selection. There is evidence that supports the purging effect in snails [14] and flour beetles [15]. Experimental evolution of *Tribolium* flour beetles, Indian meal moths and bulb mites has shown that populations evolved under high levels of sexual selection are more resistant to stressors and can take longer to become extinct, implying that they carry fewer deleterious recessives [12,15–18]. Meta-analysis has similarly shown that population fitness often increases when sexual selection on males is high [19].

Conversely, it has been proposed that high levels of sexual selection can increase genetic load by increasing the frequency of potentially deleterious alleles [20], perhaps by increasing genetic drift [21] or promoting sexual conflict [22] or other forms of inter- or intra-locus conflict [23]. In yeast, for example, it has been demonstrated that high sexual selection impairs the ability of a population to adapt to novel environments [24]. Sexual selection can drive exaggerated male traits beyond their viability optimum, and this could also decrease female fitness if there is a constraint on sex-biased trait expression or the trait is directly female harming [25]. Such sexual conflict occurs when alleles increase the fitness of one sex to the detriment of the other, and thereby increase the genetic load of a population [26]. The effect of such conflict may offset any purging effects from sexual selection and lead to a lower mean fitness in the population, as has been demonstrated both theoretically [27] and experimentally in *Drosophila melanogaster* [28–30]. Furthermore, some studies have found no effect of sexual selection on the level of deleterious mutations in either direction, rendering it relatively inconsequential for population fitness [31,32]. Ultimately, it is likely that how effectively sexual selection can purge deleterious mutations is dependent on multiple factors including environmental, social and demographic conditions [33], the strength of sexual selection, how closely sexual selection and viability optima align and the relative rates of deleterious versus advantageous mutations.

The importance of sexual selection in the mating system of *D. pseudoobscura* has been well characterized. Females are naturally polyandrous, mating with several males over their lifetime [34]. A long-running experimental evolution study manipulated sexual selection intensity by rearing lines under elevated polyandry (six males and one female) or strict monogamy (one male and one female) for approximately 200 generations [35], and phenotypic and some genomic changes between the lines have been distinguished. For example, Debelle *et al*. [36] found that heightened competition in the high sexual selection lines altered the song of the male, reducing the inter-pulse interval and increasing the number of pulses per second, potentially as an honest signal of quality. Crudgington *et al*. [35,37] found that males evolved in high sexual selection lines had larger accessory glands and were better able to manipulate females by preventing them from remating presumably via accessory gland proteins, likely increasing sexual antagonism and female harm. In addition to sexually selective traits, Garlovsky *et al*. [38] found that life-history traits also differed between the two lines, with individuals in heightened sexual selection experiencing longer developmental time, and reduced desiccation and starvation resistance compared with individuals from monogamous lines. This suggests a trade-off between sexual and non-sexual traits that reduce the overall condition of the individuals under elevated sexual selection. Previous studies of their genomic divergence have all involved pool-seq approaches and have found evidence of stronger selection in polyandrous compared with monogamous treatments, leading to particularly low levels of nucleotide diversity and strong signatures of adaptation on the third and X chromosome [39]. Wiberg *et al*. [40] found significantly lower nucleotide frequency and hotspots containing single nucleotide polymorphisms (SNPs) that show consistent allele frequency differences, especially on the third and X chromosomes and a higher fixation index (*F*_st_)between treatments on the X. Taken together, this work suggests a high level of phenotypic and genomic divergence following manipulation of sexual selection.

Here, we have completed individual-based DNA sequencing of flies from these experimentally evolved sexual selection lines i.e. those evolved under elevated polyandry (E) and enforced monogamy (M). We aimed both to clarify the genomic location and extent of signatures of selection in the genome. Further, we inferred different measures of mutational load (the genetic load of derived mutations between our lines); mean derived non-synonymous mutations (dN) of a population is an established method of gauging mutational load when mutations are additive and deleterious or neutral, and has been demonstrated to be a more accurate and robust proxy of load compared with other widely used metrics such as (dN/dS) [41]. We predicted that mean dN would be lower in the elevated polyandry lines if sexual selection was purging deleterious mutations due to purifying selection. We also compared the distribution of fitness effects (DFE) [42,43] of segregating deleterious loci to further assess the role of sexual selection in potentially purging deleterious loci from the populations. Finally, we also quantified transposable elements (TEs) between the two lines. TEs may be another signal of genetic load as they are likely to replicate more successfully when the host is stressed or otherwise in poor condition [44]. To our knowledge, this is the first study of TE load and sexual selection.

## 2. Results

A summary of the results below that support and oppose our predictions can be found in table 1.

**Table 1.** The predictions that we made in this study and the evidence that we found from our analyses to support or oppose each.

prediction	support	opposition
genomic divergence is larger at hotspots containing genetic targets of sexual selection	— higher levels of *F*_st_ at the end of chromosome 3 and throughout the X chromosome, regions previously found to contain genetic targets of sexual selection	
positive selection is stronger under elevated sexual selection	— significantly lower levels of π in E lines across the X chromosome	— no difference in π at the end of chromosome 3 between lines — significantly lower *ρ* in M lines at XR and no significant difference in *ρ* between lines anywhere else
TEs are more efficiently purged under elevated sexual selection	— significantly fewer TEs in E lines overall, and specifically in chromosome 3	— no significant difference between treatments in the X chromosome
purifying selection is greater under elevated sexual selection	— fewer alleles of deleterious effect between G85 and G200 in E lines	— no significant difference in mutational load between sexual selection treatments — no significant difference in the correlation between *ρ* and π between lines

### (a) Genomic divergence

**Figure 1.**
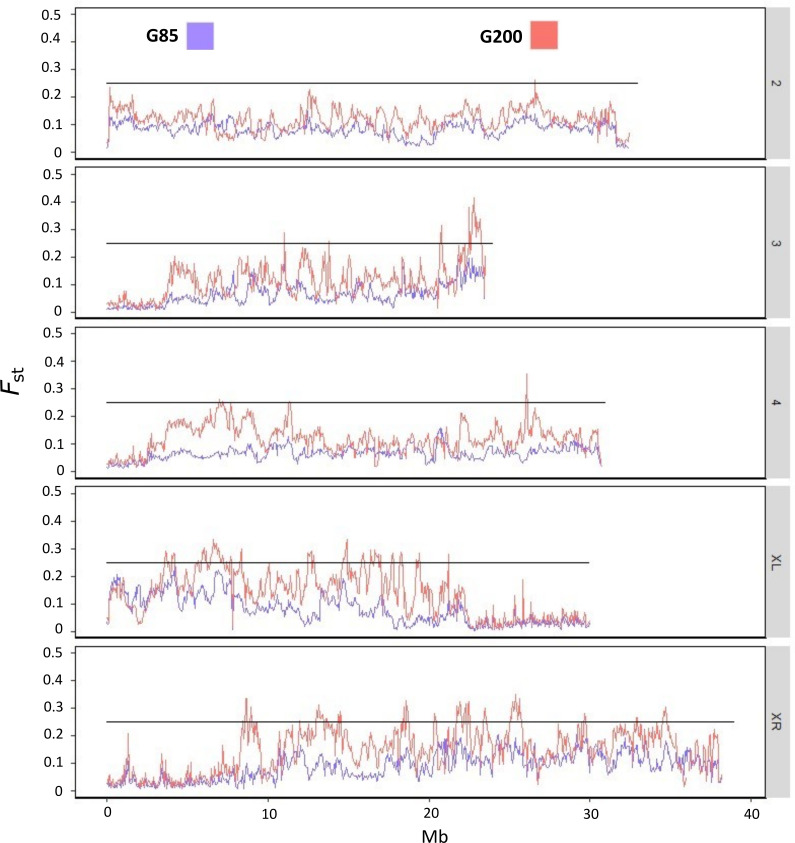
*F*_st_ of chromosomes 2, 3, 4 and X (split into XL and XR) between ‘Enhanced Polyandry’ and ‘Enforced Monogamy’ from generations 85 (blue) and 200 (red) of the sexual selection evolution experiment. Black horizontal bar shows *F*_st_ = 0.25. Individuals from each treatment and timepoint were sequenced and pooled into treatment. *F*_st_ analysis was carried out on 10 kilobase (kb) windows.

We would expect large amounts of genomic divergence between M and E lines localized at the X and third chromosomes, in agreement with previous studies [39,40]. *F*_st_ measured on individuals across two timepoints (figure 1) showed that *F*_st_ increased between generation 85 (G85) and generation 200 (G200) to produce particularly high peaks of divergence (*F*_st_ > 0.25) at the end of chromosome 3 between 22.5 and 23.5 megabases (Mb) and throughout the X chromosome. We also found a previously unidentified section in chromosome 4 that increases dramatically in *F*_st_ between G85 and G200 spanning the region approximately 3.65–13.65 Mb.

**Figure 2.**
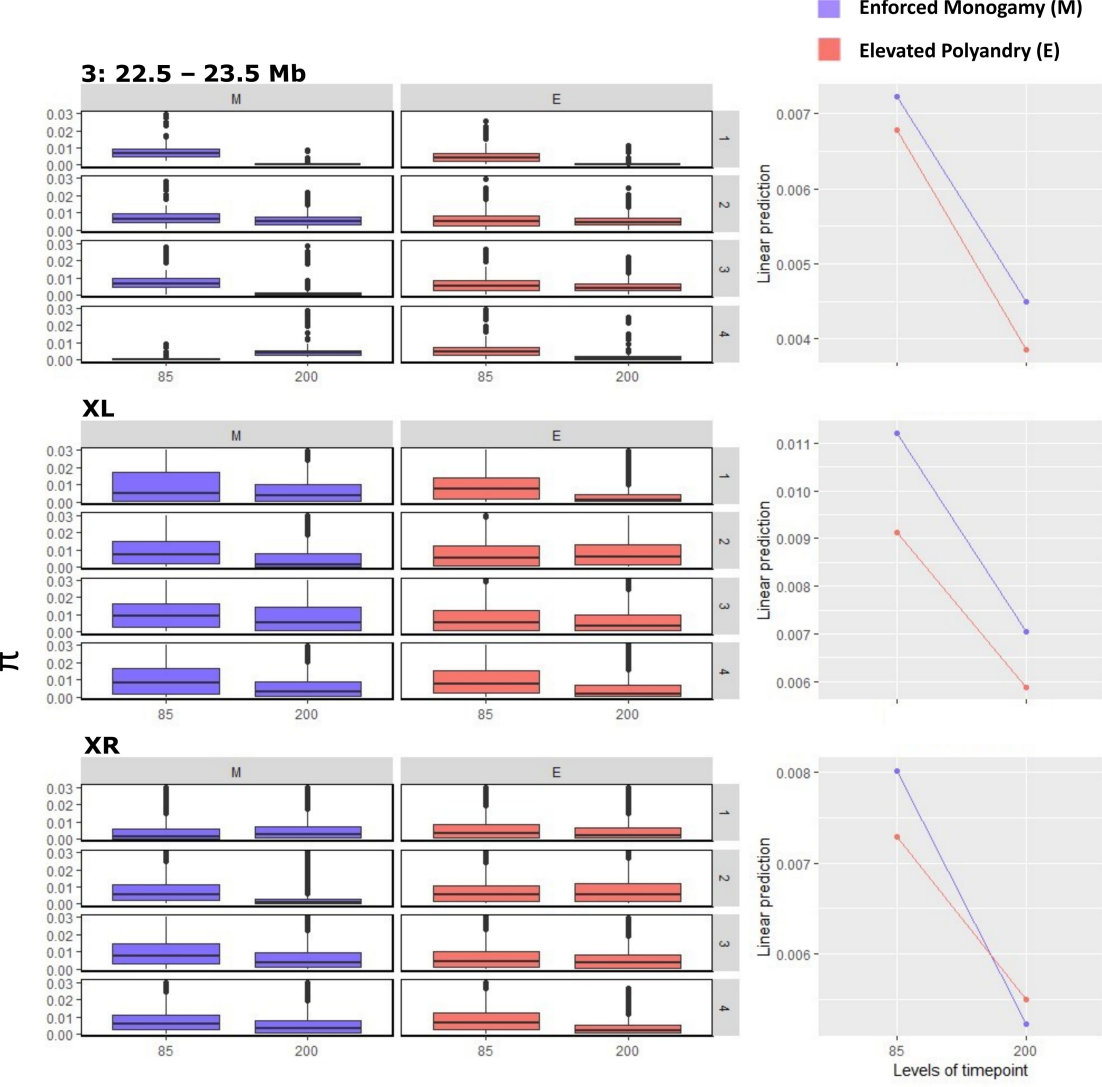
Levels of *π* at chromosomes XL, XR and 3:22.5−23.5 Mb, i.e. regions of greatest genomic divergence. On the left-hand side, *π* in each region is displayed as boxplots for each replicate (side grey boxes) and treatment (Enforced Monogamy (M) (blue) and Elevated Polyandry (E) (red)) for generation 85 and 200. On the right-hand side are estimated marginal means of *π* for each timepoint and treatment averaged over replicates.

While *F*_st_ is not independent of nucleotide diversity (*π*), large reductions in *π* alongside increased *F*_st_ might indicate strong selection in those areas. *π* was significantly lower between G85 and G200 in chromosome 3:22.5−23.5 Mb (*t*_1554_ = 9.277, *p* < 0.001), chromosome XL (*t*_46967_ = 45.781, *p* < 0.001) and chromosome XR (*t*_59602_ = 39.221, *p* < 0.001), but there was a variation in which treatment had the biggest effect in each region (figure 2). On 3:22.5−23.5 Mb, there was no significant difference between M and E lines (*t*_1554_ = 1.794, *p* = 0.073). However, *π* was significantly lower in E lines at both timepoints on XL (*t*_46967_ = 20.023, *p* < 0.001) and XR (*t*_59602_ = 3.841, *p* = 0.001). Conversely, the reduction of *π* between the two timepoints was larger in M lines (figure 2).

Our individual sequencing approach allowed us a detailed analysis of the population recombination rate (*ρ*). We found that *ρ* was significantly lower in chromosomes 3, XL and XR than 2 and 4 but only differed significantly between lines, being lower in M lines, in XR (*F*_9, 25229_ = 107.2, *p* < 0.001) (electronic supplementary material, figure S2). To further explore the effect of selection and *ρ,* we examined if *π* was positively correlated with *ρ; ρ* and *π* were positively correlated in all chromosomes for each line indicating a role for background selection in areas of low *ρ* and *π*. However, we saw no significant difference in the strength of that correlation between any of the lines suggesting that neither line is purging deleterious alleles more effectively (electronic supplementary material, figures S3 and S4).

We investigated a finding by Wiberg *et al*. [40] which suggested that *ρ* was higher in the hotspot at the end of chromosome 3 and in M lines. We found that *π* was significantly lower in this hotspot compared with the chromosome average for both lines and significantly higher in M lines than E lines (*F*_3, 3963_ = 37.48, *p* < 0.001). There was no significant difference of *ρ* between the hotspot and genome average in either line nor was there a significant difference in *ρ* within this hotspot between the two lines (*F*_3, 3963_ = 0.917, *p* = 0.432), indicating that there is no linkage in this hotspot (electronic supplementary material, figure S5).

For the jump in *F*_st_ from G85 to G200 in chromosome 4:3.65−13.65 Mb, we looked for evidence of hitchhiking but found no significant reductions in π (**M**: *F*_1, 3704_ = 0.193, *p* = 0.66; **E**: *F*_1, 3704_ = 0.0001, *p* = 0.992) or *ρ* (**M**: *F*_1, 3630_ = 2.357, *p* = 0.125; **E**: *F*_1, 3703_ = 0.044, *p* = 0.835) between this jump and the chromosome 4 average in either line which may have otherwise indicated a selective sweep (electronic supplementary material, figure S6). There is also no known inversion that spans this region.

### (b) Mutational load

**Figure 3.**
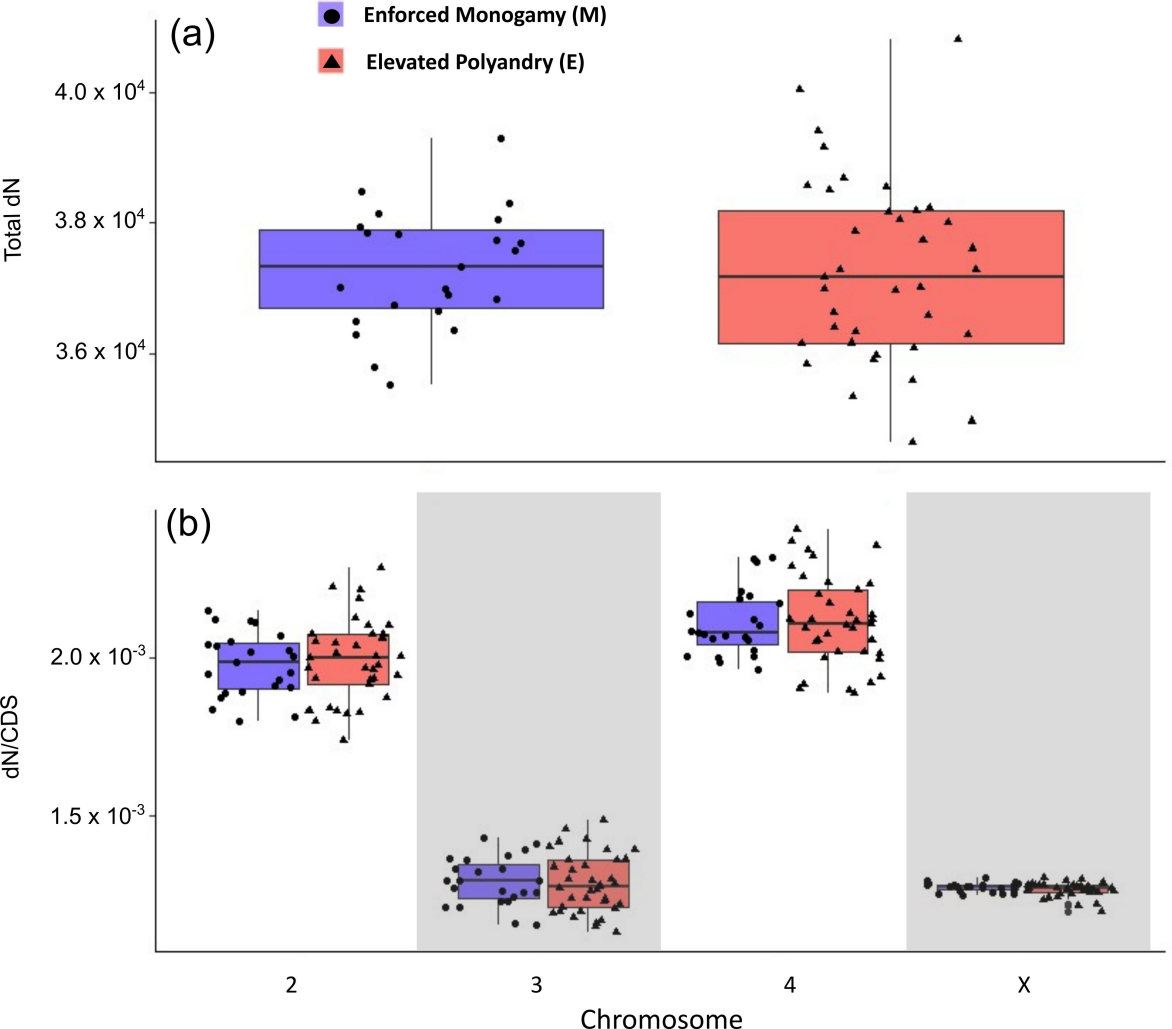
(*a*) Total number of derived non-synonymous mutations across the entire genome for samples at generation 200 in ‘Enforced Monogamy’ (M) (blue) and ‘Elevated Polyandry’ (E) (red) treatments. Normality of data was confirmed for both treatments (M: W = 0.985, *p* = 0.970; E: W = 0.982, *p* = 0.803) and variance of dN was significantly lower in M than E lines (*F*_1, 58_ = 4.305, *p* = 0.043). (*b*) Mean number of derived non-synonymous mutations per coding sequence base pair (dN/CDS bp) for each major chromosome. Boxplots show range, median, interquartile range (IQR) and 1.5 × IQR whiskers.

Taking dN as a metric of mutational load, we would expect fewer dN in E lines under strong purifying selection. Overall, no significant difference in the mean number of derived non-synonymous alleles was found between low sexual selection and high sexual selection lines across the genome (*F*_1, 58_ = 0.004, *p* = 0.953) (figure 3). Across both treatments, the number of derived non-synonymous mutations per coding sequence base pair (dN/CDS) differed significantly between chromosomes after 200 generations (*F*_7, 232_ = 532.9, *p* < 0.001), with dN/CDS lower on the X and third chromosomes (figure 3), so the X and 3 chromosomes have lower mutational load by this measure. Pairwise comparisons showed no difference between M and E lines on any chromosome (**Chromosome 2**: *t*_232)_ = −0.546, *p* = 0.586; **3**: *t*_232_ = 0.207, *p* = 0.836; **4**: *t*_232_ = −0.227, *p* = 0.821; **X**: *t*_232_ = 0.310, *p* = 0.757). There was considerably more between replicate variation in E lines than M lines (electronic supplementary material, figure S7). We also looked for dN that consistently fixed in all replicates of each line, which would suggest treatment-based fixation due to selection as opposed to drift. We found that consistently fixed dN was similar between lines although slightly lower in E lines within each chromosome, especially chromosome 3 (electronic supplementary material, figure S7).

### (c) Transposable elements

**Figure 4.**
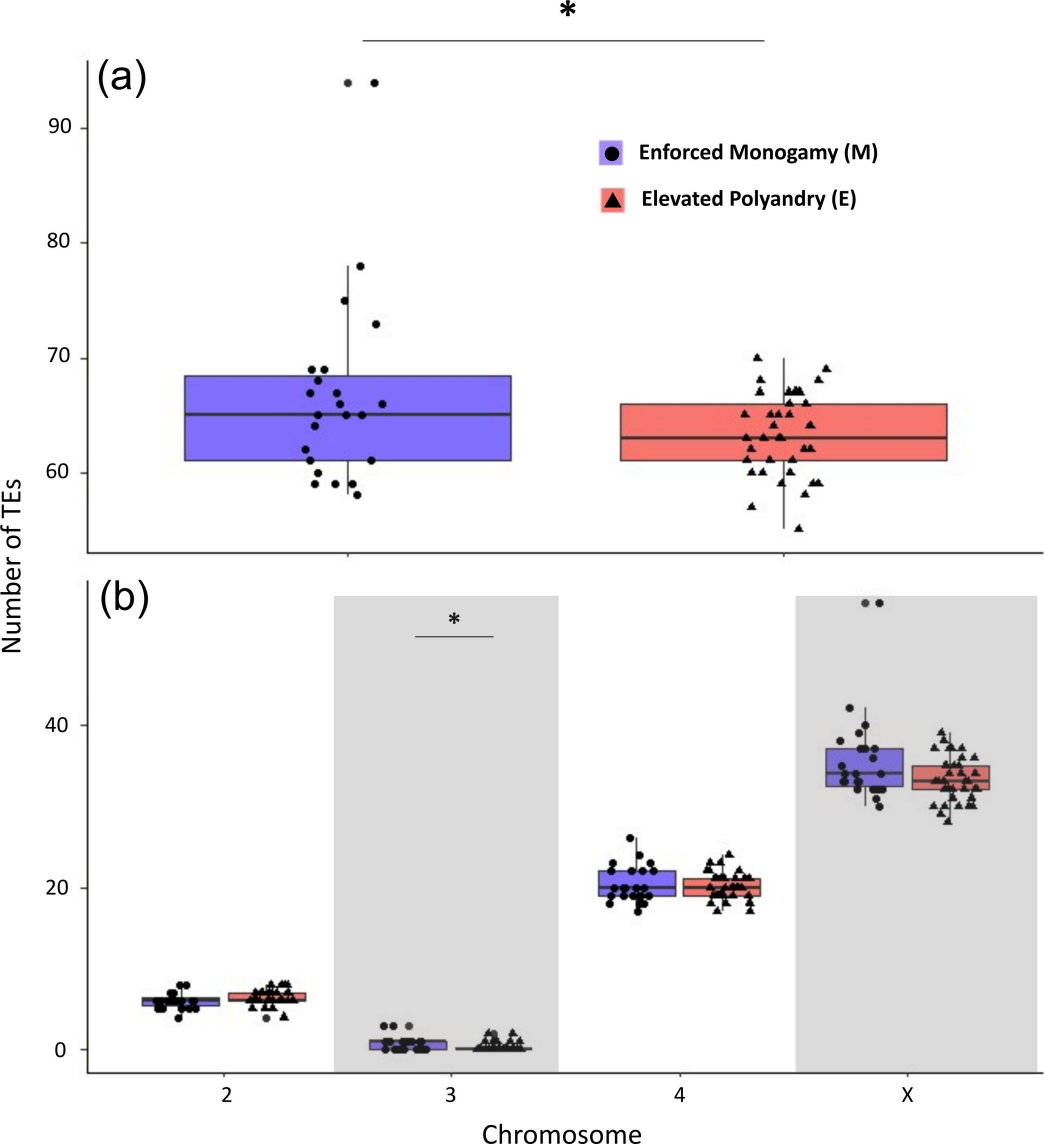
Number of identified transposable element (TE) sequences found in Enforced Monogamy (M, blue) and Enhanced Polyandry (E, red) lines. (*a*) Overall number of TE sequences found in each sequenced individual in each line. This is further broken down into (*b*) the number of TE sequences found in each chromosome from each sequenced individual and (*c*) the number of TE sequences identified from different TE families from each sequenced individual. Boxplots show range, median, interquartile range (IQR) and 1.5 × IQR whiskers. Asterisk between experimental evolution lines indicates *p* < 0.05.

We expected to see a difference in TEs if there is a difference in stress or condition perpetuated by differing sexual selection regimes. We detected significantly more TE sequences in M lines than E lines (*F*_1, 58_ = 4.664, *p* = 0.035) (figure 4). We also found a significant interaction between treatment and chromosome on TE number (*F*_3, 232_ = 4.602, *p* = 0.004), with significantly more TEs on M lines found on chromosome 3.

### (d) Distribution of fitness effects

#### (i) Deleterious effects

**Figure 5.**
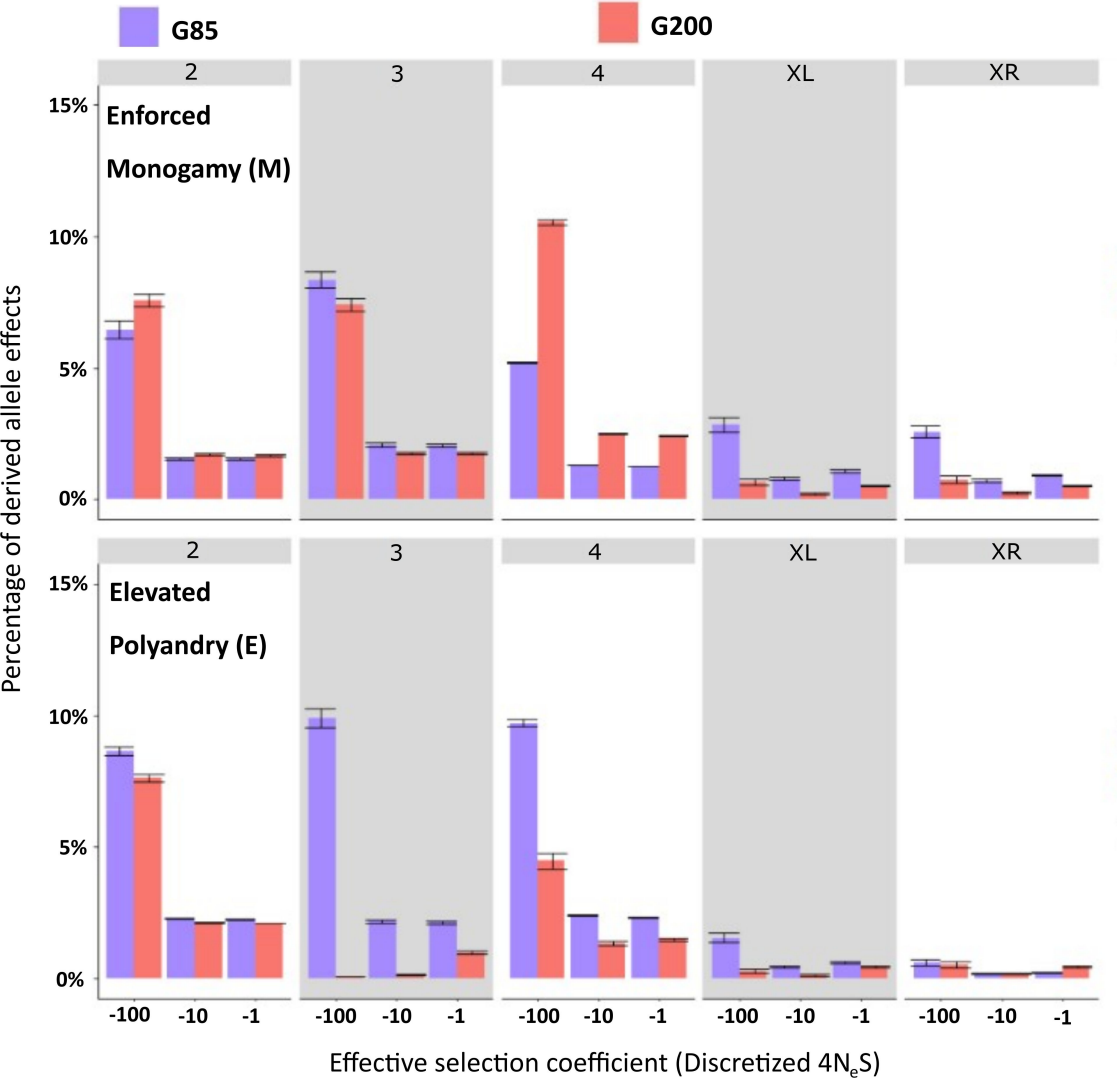
Distribution of deleterious fitness effects (DFE) for the *Drosophila pseudoobscura* evolved under ‘Enforced Monogamy’ (top) and ‘Elevated Polyandry’ (bottom) at each chromosome. Shown are the DFE at generation 85 (blue) and generation 200 (red). Bars show the mean proportion of derived allele effects and 95% confidence intervals (2 s.d. from the mean) at each respective discretized selection coefficient calculated from 500 bootstrapped site frequency spectra profiles. Discretized selection coefficient, with a lower value indicating a stronger deleterious effect, is shown on the *x*-axis. The *y*-axis is the percentage of derived allele effects at the corresponding discretized fitness group. Chromosome numbers and X arms are indicated within the grey boxes.

Here, we aimed to see how effectively each line could purge or maintain derived alleles of beneficial or deleterious effect at two directly measurable timepoints, using the reference genome as an approximation of the initial allele frequency distribution. If purging has occurred, then we expected deleterious alleles to have diminished in frequency, especially in the E lines. Between G85 and G200, the proportion of deleterious allelic effects in E lines largely reduced on chromosomes 3 and 4, consistent with more efficient purging selection. However, they increased on chromosome 4 within M lines between G85 and G200 (figure 5). Also, on both of the X chromosome arms, the proportion of deleterious allelic effects decreased more within M lines than E lines between G85 and G200, i.e. M lines were more effective at purging deleterious alleles despite the lack of sexual selection, so any purging effect on the X chromosome may have been due to more efficient natural selection during the experiment.

#### (ii) Beneficial effects

**Figure 6.**
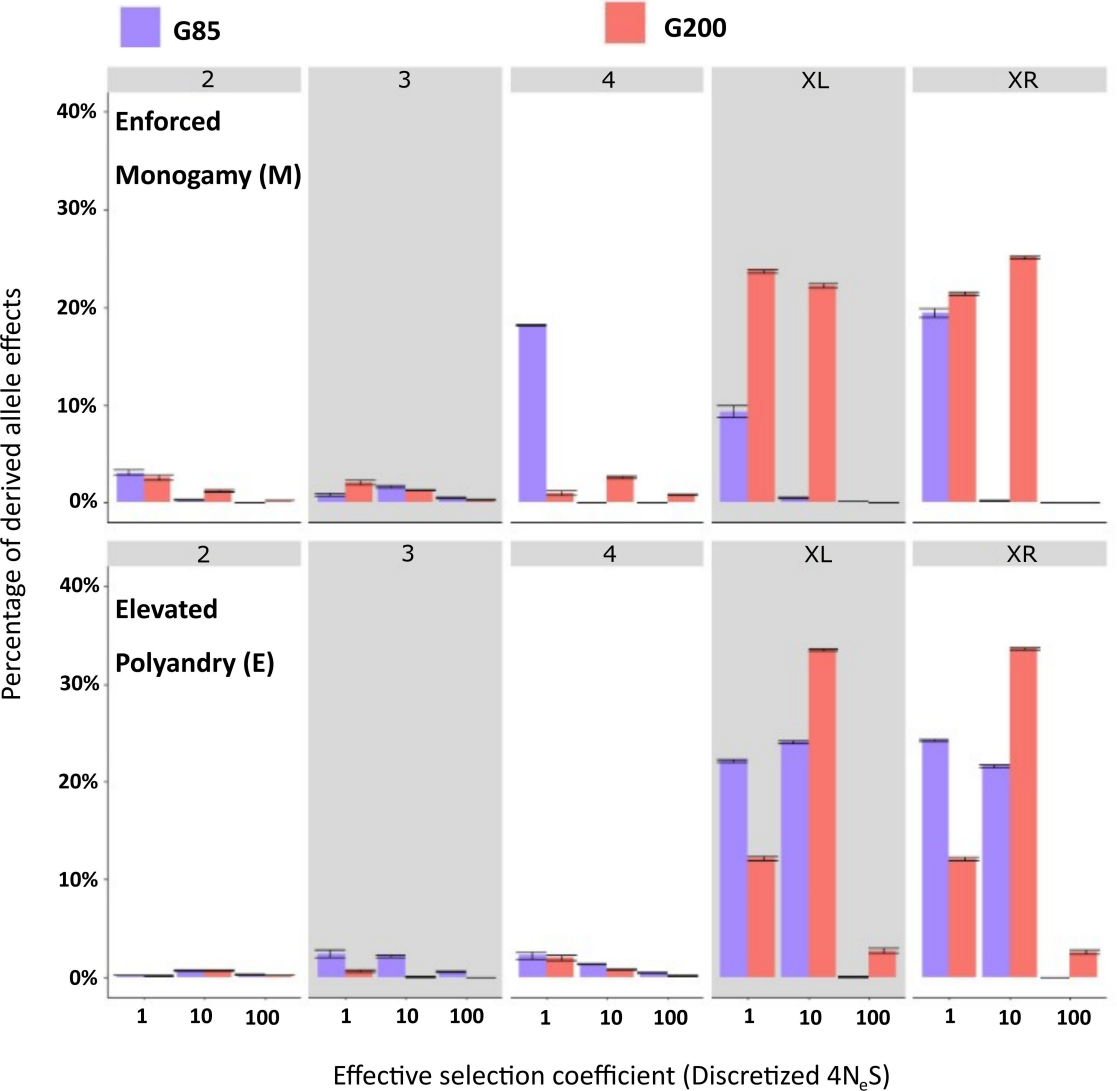
Distribution of adaptive fitness effects (DFE) for the *Drosophila pseudoobscura* evolved under ‘Enforced Monogamy’ (top) and ‘Elevated Polyandry’ (bottom) at each chromosome. Shown are the DFE at generation 85 (blue) and generation 200 (red). Bars show the mean proportion of derived allele effects and 95% confidence intervals (2 s.d. from the mean) at each respective discretized selection coefficient calculated from 500 bootstrapped site frequency spectra profiles. Discretized selection coefficient, with a higher value indicating a more beneficial effect, is shown on the *x*-axis. The *y*-axis is the percentage of derived allele effects at the corresponding discretized fitness group. Chromosome numbers and X arms are indicated within the grey boxes.

Changes in beneficial allelic effects between G85 and G200 were less dramatic on the autosomes, but there was a peak on chromosome 4 in the monogamous lines at G85 which is fixed or lost by G200 (figure 6). The X chromosome showed much more evidence for advantageous alleles, but these were seen in both E and M lines so these effects may reflect alleles that are partially recessive increasing in frequency over the course of the experiment under both natural and sexual selection (figure 6). For the full DFE statistics, see electronic supplementary material, S8.

## 3. Discussion

Sexual selection has often been argued to purge deleterious loci but, in contrast, alleles under such selection could involve a trade-off between mating success and viability in males, or sexual antagonism between any ‘good gene’ effects could counterbalance sexual selection benefits by increasing female harm [27,30]. Our results showed that the overall mutational load did not differ between high and low sexual selection treatments. However, there was evidence for both purging of deleterious alleles and fixation of beneficial alleles in response to sexual selection, but this differed over the course of the experiment and between different chromosomes. Hence, and perhaps unsurprisingly, complex interactions between purifying selection and natural and sexual selection appear to occur across the genomic landscape. Unexpectedly, one of the more pronounced effects we detected was fewer TEs in lines subject to strong sexual selection.

The largest regions of genomic divergence were located on the third and X chromosomes. This agrees with previous pool-seq analyses of these lines, which found hotspots of divergence in the same regions [39,40]. We found a significant decrease in genetic diversity and across timepoints in each line, which is a strong indication of positive selection within these regions. Using our own data, we examined a hotspot at the end of chromosome 3 that had been previously found to show surprisingly high rates of recombination within these sexual selection lines [40]. Here, we found no significant difference in the rates of recombination compared with the chromosome average or between lines; however, we did see significantly lower levels of *π*, especially in the E lines, which is what we would expect under positive selection. Interestingly, changes in *π* over generations did not occur in the same manner for each treatment at each region. Both treatments showed a similar reduction of *π* at the end of chromosome 3, but across chromosome XL, *π* was significantly lower in E lines at both timepoints, and the reduction of *π* was more pronounced in M lines across XR. This demonstrates that the different mating regimes, monogamy and elevated sexual selection, affect different areas of the genome separately and supports work by Barata *et al*. [39] who found greater signatures of adaptation across XR in M lines, and across 3 and XL in E lines. Our individual-based sequencing allowed for a high resolution of recombination compared with previous work on these lines. Areas of high *F*_st_ and low *π* were positively correlated with low levels of *ρ*, indicating a role for background selection. However, there was no difference in the strength of this correlation between lines in any chromosome, suggesting that neither line is more efficiently purging deleterious alleles.

Barata *et al*. [39] also found a small signature of adaptation in the region of chromosome 4 between 3.65 and 13.65 Mb where we find high *F*_st_ between G85 and G200. As it is such a large region, we investigated the possibility of hitchhiking to explain why this region diverged so quickly. There is no known inversion that spans this region and after removing the centromere from analysis we found no difference in π or *ρ* between this hotspot on 4 and the chromosome average in either line. This suggests an absence of strong linkage. This jump is therefore puzzling but may be due to drift or balancing selection acting on different alleles in response to differing sexual selection regimes.

Overall, we found little evidence to support the notion of sexual selection purging deleterious loci in measures of mutational load. There was no difference in total genomic dN seen between treatments; however, variance was significantly higher in E lines as driven by higher variation between the individual replicates. This may underly the higher evolutionary potential of sexual selection to be adaptive or deleterious. There were significant differences in dN/CDS across chromosomes. dN/CDS was much lower on the X and third chromosomes, which again fits with our pattern of selection being strongest on these chromosomes. We would expect purifying selection to be more efficient on the X chromosome, as hemizygosity renders recessive X-linked genes more visible to selection [45]. But our analyses suggest that these chromosomes also show more efficient purging selection independent of sexual selection. An important caveat here is that using total dN to measure load is a method that assumes mutations are additive and deleterious or neutral [41]. The X chromosome did not seem to fulfil those assumptions here as the shape of the DFE showed most derived mutations are of beneficial allelic effects; therefore, interpretations of mutational load from this method should be treated with some caution. Regardless, there was evidence here that the X chromosome is being shaped by multiple processes, some purging but also evidence of both sexual and regular adaptive effects independent of sexual selection (i.e. as positive selection and similar levels of dN were also seen in M lines).

While overall mutational load was similar between M and E lines, there were distinct differences in the shape of the DFE. This occurred most dramatically in E lines between G85 and G200, with reduced deleterious allelic effects on chromosomes 3 and 4 but also a smaller proportion of beneficial allelic effects. Conversely, the shape of the DFE in M lines remained relatively consistent across the duration of the experiment and even increased the proportion of deleterious effects on chromosome 4. This is the clearest evidence for the expected purging effect of sexual selection in our study, but it obviously does not predominate the responses uncovered. We do not have data on the specific loci that were being selected for, but it would be of great interest to see if these loci align with loci associated with differential expression and genomic divergence between these lines, as reported by Veltsos *et al*. [46] and Wiberg *et al*. [40], respectively.

One clear and unexpected result that we saw was that significantly more TEs were found within M lines, especially on the more divergent chromosome 3. This suggests that sexual selection can successfully help remove potentially harmful TEs. If TEs confer a deleterious effect in the genome, as is predicted [47], then there may be purging of TEs when sexual competition is high. In *D. pseudoobscura,* polyandry has been shown to increase as an adaptive response to reduce other selfish genetic elements such as segregation distorters [48]. Perhaps increased remating can also counter the spread of TEs better than monogamy.

We note that laboratory adaptation was not tested in these lines. While significant differences between the lines have been observed, both lines share some similar patterns in the direction of their evolution between G85 and G200. Some of this may be due to laboratory adaptations affecting both lines similarly which may be hard to untangle from the effect of sexual selection between timepoints. Some of the G200 samples were also from G165 which might have added noise to the results. While this is likely to have inflated estimates of variation, previous analysis [39] has shown that the biggest signatures of selection occur by generation 100; therefore, we would expect differences between G165 and G200 to be comparatively small. Finally, we have used MV2−25 as our reference genome rather than the ancestral population of the experimental evolution lines as this base population is not available. This could have the effect of overinflating estimates of dN as some variants will have been fixed in both populations prior to the experiment. To account for this, we filtered out the variants fixed within both lines. However, this is a somewhat crude method of correction, and subtle but potentially significant differences in dN between the lines may not have been picked up.

## 4. Conclusion

Here, we show that altering the mating system by modifying the strength of sexual selection has complex genomic consequences. Purging deleterious alleles from a population should help to reduce extinction risk [49] but, in contrast, high sexual conflict or sexual-natural selection trade-offs or increased drift could lead to a significant genetic load reducing population fitness. Sexual selection can alter the distribution of fitness effects from segregating deleterious loci in a way that could have complex consequences for a population in a rapidly changing environment. Our results find that sexual selection can be effective in purging deleterious allelic effects, but ensuing high sexual conflict may limit the spread of potentially adaptive alleles. Despite these complex interactions, our lines show similar mutational load between high and low sexual selection lines. This complexity may go some way to explaining the contradictory reports of sexual selection and population fitness and help to reconcile predictions on population persistence with high sexual selection (e.g. [15,50]) and observations of higher extinction rates of sexually selected species [51,52].

## 5. Material and methods

### (a) Treatments

*Drosophila pseudoobscura* is a polyandrous species, and it is estimated that a female will mate with 2−3 males in her lifetime [53]. Therefore, sexual selection was manipulated by controlling the number of males available for the female to mate with. The treatments were ‘Enforced Monogamy’ (M) which consisted of one female kept with one male, effectively eliminating sexual selection by removing competition between males, and ‘Elevated Polyandry’ (E) which consisted of one female with six males, thereby exacerbating competition between males and intensely increasing sexual selection pressure. Males were kept with females for 5 days in each generation before being separated for the oviposition period. Differences in the effective population size may bias DFE comparisons, with low effective population sizes being associated with a higher number of deleterious mutations due to drift or inbreeding [54]. In this design, monogamous lines would have a naturally lower effective population size; therefore, more monogamous females were used compared with the polyandrous treatment to keep the effective population size (*N*_e_) consistent (for full details of the experimental evolution rearing protocol see [35]). Previous analyses on these lines have confirmed that overall effective population size was fairly large in each treatment and not consistently significantly different between lines [39,55].

We sequenced individual males from each of the four replicates of the M and E treatments at two timepoints—generation 85 (G85) and generation 200 (G200). Sample sizes for each replicate were: **G85–M1**: 2, **M2**: 7, **M3**: 10, **M4**: 6, **E1**: 10, **E2**: 8, **E3**: 8, **E4**: 12; **G200–M1**: 8, **M2**: 2, **M3**: 4, **M4**: 9, **E1**: 9, **E2**: 9, **E3**: 9, **E4**: 10, giving a total number of **G85–M**: 25, **E**: 38; **G200–M**: 23, **E**: 37. Some of the G200 samples were degraded so 22 of the 62 ‘G200’ males were instead sequenced from generation 165, which was the closest timepoint to G200 from which samples were available. For the full DNA extraction protocol, see electronic supplementary material, S1.

### (b) Genomic sequence processing

Illumina sequencing at 30× coverage was carried out by Novogene. The range of the read quality was: raw reads = 23704758– 51288002, effectiveness = 98.67–99.4%, error = 0.03%, Q20 = 96.16–97.79% and Q30 = 90.54–93.84%. Raw reads were assessed for quality using Fastqc [56], pre- and post-quality trimming. Reads were quality (Q30) and adaptor trimmed using trimmomatic v.0.39 [57]. Trimmed reads were then mapped to the MV2-25 *D. pseudoobscura* assembly as a reference (https://www.ncbi.nlm.nih.gov/assembly/GCF_009870125.1/) using bwa-mem2-2.0 [58]. Duplicates in the mapping bam files were marked using picard-2.18.29-0 [59]. GATK HaplotypeCaller was used to call variants [60]. The resulting variant call file (VCF) was interrogated using VCFtools [61], and replicates were combined into treatments and timepoints. Scripts can be found at: https://github.com/peterthorpe5/Drosophila_pseudoobscura_selective_sweeps.

### (c) Descriptive statistics

VCFtools was used to calculate *π* (*--window-pi*) in 10 kb windows, and *F*_st_ (*--weir-fst-pop*) in 50 kb windows across each chromosome. LDHat was used to calculate recombination rate using the LDhat_workflow pipeline [62,63] across each chromosome, these were then averaged across 10 kb windows in R v.4.1.3 [64] to reduce noise and make it directly comparable to our estimates of *π*. Replicates were combined into polyandrous or monogamous treatments at each timepoint prior to *ρ*, *F*_st_ and TE analyses.

We investigated the levels of π in areas of high *F*_st_, fitting a linear model that used *replicate*, *timepoint* and *treatment* as predictors. Linear models with *genome region* as a predictor variable were used to analyse differences in *ρ* between hotspots and the genome average for each treatment, and with *chromosome* and *treatment* as predictor variables to analyse differences in *ρ* between treatments. A Spearman’s rank test was used to analyse strength of the correlation between *π* and *ρ*, and a *z*-score was calculated from Fisher *z*-transformed correlation coefficients to compare the strength of the correlation between treatments. Specific to these analyses, the centromeric region was removed from each chromosome in order to reduce biologically irrelevant noise. All statistical analysis was carried out in R v.4.1.3.

### (d) Mutational load

We counted the total number of dN across the genome and per chromosome from our samples as compared with the reference genome. As we did not have any base population sequences to use as a reference, common variants present in every sample were removed. This was to avoid overestimation of dN as these variants were likely to be already fixed in the original base population. For each chromosome, we divided the dN by the coding sequence length to give the average number of dN/CDS. As an addendum, we also compared the numbers of dN that were present in all samples, and therefore ‘fixed’, within each treatment. Annotated VCF files identifying variant alleles compared with the annotated MV2−25 reference were produced by ANNOVAR (ANNOtate VARiation) [65] and non-synonymous changes comprising nonsense and missense mutations, as well as indels, were quantified for each chromosome in each sample. Total dN and dN/CDS between treatments were graphed and analysed using a linear model with *treatment* and *chromosome* as our predictors. For post hoc pairwise comparisons, *emmeans* with Tukey adjustment was used, normality was tested using a Shapiro–Wilk test and variance was analysed using a Levene’s test in R v.4.1.3 [64].

### (e) Transposable elements

TE sequences were identified using PoPoolation TE2 (*-jar popte2-v1.10.04.jar identifySignatures, frequency, pairupSignatures & stat-coverage*) [66] with *D. pseudoobscura* reference sequences obtained from a study by Hill & Betancourt [67]. Differences between sexual selection lines were analysed using a generalized linear model with quasi-Poisson family (to account for under-dispersion in our count data) and log link; *TE number* was the response variable and the *sexual selection treatment* was the predictor variable. To test for differences in the TE number between chromosomes, we again used a generalized linear model with a quasi-Poisson family and log link; *TE number* was the response variable, and *chromosome* and *sexual selection treatment* were the main predictor variables, as well as *chromosome* × *sexual selection treatment* as an interaction term.

### (f) Calculating DFE estimates

Due to low sample size in some replicates, individual sequencing data from all replicates were pooled into ‘M’ and ‘E’ treatments for each timepoint for analysis. For each treatment, an estimated site frequency spectra (SFS) with known ancestral state (unfolded SFS) were constructed on dS (synonymous) and dN (non-synonymous) mutations using easySFS [68,69]. Projection values were chosen using the --*proj* flag that maximized the number of segregating sites in each sample and chromosome while balancing the sample size number, in clear terms this meant choosing a projection value slightly higher than that which contained the maximum number of segregating sites but still contained a number of segregating sites close to the maximum. X chromosomes were processed as haploid using the --*ploidy 1* flag as only males were used in this study. A DFE profile on the dN SFS was constructed with polyDFE [70] with the dS SFS used as the neutral baseline. polyDFE uses a maximum-likelihood approach to model inferred fitness from the segregating allele frequencies (via the SFS) of non-synonymous sites, using the neutral sites as a baseline. From this model, a scaled fitness coefficient to new mutations under selection is assigned. It assumes that fixation or loss of neutral mutations results purely from drift, that new mutations arise randomly and independently as determined by the mutation rate of each site and that new mutations are not inherently fit or unfit. Here, we supplied the SFS from both lines at the two timepoints to look at how the DFE changes over time and sexually selective pressure compared with the reference genome, which we used as an approximation of initial allele frequencies owing to our lack of a base population. Multiple models and parameters were tested, and the best model was chosen (*C + r + ε*_an_) using the Akaike Information Criteria in the ‘*compare.model*’ R function included in polyDFE. This assumes a mixture of gamma and exponential distributions and includes a series of nuisance parameters (*r_i_*) to account for potential SFS distortion and parameters accounting for polarization error (*ε*_an_) [70]. Upon selecting the best fitting model for the data, the SFS for each replicate was bootstrapped 500 times, and polyDFE was run on each to create a range of DFE profiles for each replicate. The mean and 95% confidence intervals (± 2 s.d. from the mean) were obtained for each treatment in each chromosome at G85 and G200 using the ‘*SummarySE’* function in R v.4.1.3 [64]. polyDFE quantifies the log-scaled selection coefficient of each derived allele and categorizes them into discrete 4*N*_e_*S* values (‘<−100’, ‘<−10’, ‘<−1’, ‘0’, ‘>1’, ‘>10’, ‘>100’) corresponding to the effect of the mutation, with a lower number representing a stronger deleterious effect and higher number representing a stronger beneficial effect.

## Data Availability

All sequence data collected and used in this study are available at the Sequence Read Archive (SRA): Accession no. PRJNA983697 [71] https://www.ncbi.nlm.nih.gov/sra/PRJNA983697. Code used for this study, and Excel spreadsheets containing processed count TE and dN data are available at [72,73]. Supplementary material is available online [74].
